# Pharmacological randomized controlled trials in acute respiratory distress syndrome mortality

**DOI:** 10.1186/cc9609

**Published:** 2011-03-11

**Authors:** C Santacruz, E Carrasco, J Wardini Dantas do Amaral

**Affiliations:** 1 Fundacion Abood Shaio, Bogota, Colombia; 2 Hospital Valladolid, Spain; 3 Erasme Hopital, Brussels, Belgium

## Introduction

Acute lung injury and acute respiratory distress syndrome are common conditions encountered in the ICU. Whether mortality has decreased over time or not, they are still many unanswered questions about the impact of pharmacological treatment on ALI/ARDS mortality.

## Methods

The objectives were to perform a review of the literature in search of the randomized control trials that asses the pharmacological impact in ALI/ARDS on all-cause mortality. We included all RCTs of pharmacological treatments in ALI/ARDS that had an impact in mortality in adults. We excluded RCTs that included patients <18 years old and animals. We also excluded trials that tested fluid therapy, mechanical ventilation, nonpharmacological treatments, antibiotics and reviews. No date or language restriction was applied.

## Results

We included 37 RCTs involving 6,303 patients in different ALI/ARDS treatment modalities: steroids (*n *= 271), enteral nutrition (*n *= 411), surfactant (*n *= 1,754), nitric oxide (*n *= 1,342), APC (*n *= 75), muscle relaxants (*n *= 340), prostaglandins (*n *= 550), NAC (*n *= 127), silvelastat (*n *= 492), rPAF-HD (*n *= 127) lisofylline (*n *= 235), rFVIIa antagonist (*n *= 214), OTZ (*n *= 215) and verapamile-procaine compound (*n *= 150).

## Conclusions

Only steroid treatment (methylprednisolone) and nutritional therapy (EPA + GLA + antioxidants) showed a trend towards reduced mortality. Other treatments were associated with reduced morbidity. However, many empirical treatments are still used in day-to-day practice.
